# Enocyanin Synergistically Enhances Sorafenib Sensitivity in Hepatocellular Carcinoma via Ferroptosis Induction Associated with p62/Keap1/Nrf2/HO-1 Pathway Inhibition

**DOI:** 10.3390/cimb48040357

**Published:** 2026-03-28

**Authors:** Mengting Tian, Jing Ma, Tingting Wei, Kunqi Meng, Yingmeng Xia, Xue Zong, Changcai Bai, Zhisheng Wang

**Affiliations:** 1 School of Pharmacy, Ningxia Medical University, Yinchuan 750004, China; 2 School of Inspection, Ningxia Medical University, Yinchuan 750004, China; 3 The First Clinical College, Ningxia Medical University, Yinchuan 750004, China; 4 School of Public Health, Ningxia Medical University, Yinchuan 750004, China

**Keywords:** hepatocellular carcinoma, HepG2, Enocyanin, sorafenib, ferroptosis, p62/Keap1/Nrf2/HO-1

## Abstract

Hepatocellular carcinoma (HCC) poses a critical threat to global health because of the scarcity of effective therapeutic approaches. Sorafenib, a first-line treatment for advanced HCC, often faces efficacy limitations due to acquired resistance. Therefore, it is urgent to explore novel and effective anti-cancer drugs and combination therapies. This study explored the anti-HCC potential of Enocyanin (Eno), a natural anthocyanin-rich extract derived from grapes, either alone or combined with sorafenib. Our findings indicated that 100 μg/mL Eno remarkably suppressed the proliferation, invasion and migration of HepG2 cells, which was related to the induction of ferroptosis characterized by increased intracellular Fe^2+^, lipid peroxidation (LPO) and Acyl-CoA synthetase long chain family member 4 (ACSL4) levels, coupled with decreased glutathione (GSH) and glutathione peroxidase 4 (GPX4). Mechanistically, Eno promoted ferroptosis which was associated with inhibition of the p62/Keap1/Nrf2/HO-1 signaling pathway. Notably, Eno (100 μg/mL) combined with sorafenib (2 μM) had a synergistic anti-tumor effect (Q = 1.47), which further enhanced the inhibition of HepG2 cell growth and metastasis, aggravated ferroptosis, and more strongly suppressed the p62/Keap1/Nrf2/HO-1 axis. In the C57BL/6 mouse subcutaneous HCC transplantation model, the combination of Eno and sorafenib showed a stronger inhibitory effect on tumor growth, reaching a 70% inhibition rate, compared to 33% with Eno alone and 55% with sorafenib alone. In summary, this study demonstrates that Eno may be a novel inducer of ferroptosis, and it has the potential to be used in the treatment of hepatocellular carcinoma. It also provides a potential combined treatment strategy for enhancing the sensitivity of sorafenib.

## 1. Introduction

Hepatocellular carcinoma (HCC) constitutes the vast majority of primary liver malignancies, characterized by high occulticity, invasiveness, and mortality, presenting a significant public health challenge [1,2]. Although considerable progress has been made in clinical management, surgical resection remains the primary therapeutic approach, while pharmacotherapy usually serves as an adjuvant intervention [3]. Among the treatment options, the first-line drugs represented by multikinase inhibitors (e.g., sorafenib and lenvatinib) have been widely used [4,5]. These drugs exert anti-proliferative and anti-angiogenic effects by inhibiting multiple tyrosine kinases (e.g., VEGFR and PDGFR) [6,7,8]. Nevertheless, these drugs generally exhibit limited efficacy, are prone to inducing drug resistance, and cause significant adverse reactions (e.g., hypertension, hand–foot skin reaction, and liver function damage), which severely restrict the long-term clinical benefits for patients [9,10]. Studies have reported that the median survival benefit of patients treated with sorafenib for tumors is usually only 2–3 months, and due to the occurrence of drug resistance, the treatment outcome is often difficult to achieve a breakthrough [11]. Therefore, the development of new strategies that can overcome drug resistance, improve efficacy and reduce side effects is still a key issue to be solved in the field of HCC treatment.

In recent years, ferroptosis, an iron-dependent form of regulatory cell death characterized by uncontrolled lipid peroxidation, has become a key mechanism affecting the efficacy of sorafenib in the treatment of HCC [12]. A large amount of evidence shows that the efficacy of sorafenib is partly due to its ability to induce ferroptosis, and the imbalance of ferroptosis signaling is closely related to the emergence of acquired drug resistance [13]. A key regulatory axis for ferroptosis is associated with the p62-Keap1-Nrf2 signaling pathway [14]. Under stress conditions, the adapter protein p62 can sequester kelch-like ECH-associated protein 1 (Keap1), thereby stabilizing the transcription factor nuclear factor erythroid 2-related factor 2 (Nrf2). This allows Nrf2 to translocate to the nucleus and drive the expression of a battery of antioxidant genes, including heme oxygenase-1 (HO-1), which collectively act to mitigate oxidative damage and counteract ferroptosis [15]. Consequently, targeting this pathway to reinstate ferroptosis sensitivity represents a promising strategy for overcoming sorafenib resistance.

Natural products, with their rich chemical diversity and multi-target capabilities, offer a valuable reservoir for discovering novel anticancer agents or chemosensitizers [16]. Notably, combining natural products with sorafenib has demonstrated enhanced antitumor efficacy in HCC models. For example, artesunate and camptothecin have been reported to synergize with sorafenib by modulating iron metabolism, reactive oxygen species (ROS) accumulation, or antioxidant systems [15,17]. Anthocyanins, a class of bioactive flavonoids abundant in grapes and other botanicals, exhibit potent anti-inflammatory, antioxidant, and antitumor properties [18,19]. Enocyanin (Eno), an anthocyanin-rich extract from grape, has been reported to have anti-inflammatory effects, regulate the composition and stability of intestinal microbiota, and promote bone formation [20,21]. However, its potential anti-HCC effects, particularly in relation to ferroptosis induction and combination therapy with sorafenib, remain largely unexplored.

This study aims to systematically evaluate the therapeutic potential of Eno in HCC, its synergistic effect with sorafenib, and the underlying mechanism. Through in vitro cell experiments and a subcutaneous xenograft tumor model in mice, the inhibitory effects of Eno alone and in combination with sorafenib on the proliferation, metastasis of HepG2 cells, and tumor growth were evaluated. Moreover, the expression changes in ferroptosis-related markers and key proteins in the p62/Keap1/Nrf2/HO-1 signaling axis were specifically detected to clarify the synergistic anti-tumor mechanism. The research results will provide experimental evidence and theoretical basis for the clinical combined treatment strategy of HCC.

## 2. Materials and Methods

### 2.1. Reagents

Eno (catalog number: HY-114336; lot number: 37221; CAS No.: 11029-12-2; Polyphenols: 32.3%, Anthocyanidins: 2.9%), Liproxstatin-1 and Sorafenib were obtained from MCE (Monmouth Junction, NJ, USA). Eno was dissolved in Dulbecco’s Modified Eagle Medium (DMEM, Gibco, CA, USA), Liproxstatin-1 and Sorafenib were dissolved in DMSO (In cell experiments, the final concentration is less than 0.1%; in animal experiments, the volume ratio is less than 5%) and filtered through a 0.22 μm filter membrane. Working solutions were serially diluted in the medium to achieve the final concentration. The stock solutions were stored at −80 °C, and the working solutions were stored at −20 °C. All solutions were thawed before use.

### 2.2. Antibodies

p62 from Abcam (Cambridge, UK); Nrf2 from Proteintech (Chicago, IL, USA); Keap1 from Proteintech (Chicago, IL, USA); HO-1 from ABclonal (Wuhan, China); GPX4 from ABclonal (Wuhan, China); Ki-67 from Abcam (Cambridge, UK); β-actin from ZSGB-BIO (Beijing, China); HRP-conjugated Goat Anti-Rabbit IgG (H + L) from Affinit (Jiangsu, China); HRP-conjugated Goat Anti-Mouse IgG (H + L) from Abways (Shanghai, China); FITC-conjugated Goat anti-rabbit/mouse IgG from ZSGB-BIO (Beijing, China).

### 2.3. Cell Culture and Treatment

The human HCC cell line HepG2 and the mouse HCC cell line Hepa1-6 (obtained from ATCC), were used in this study. Cells were routinely propagated in DMEM containing 10% fetal bovine serum (FBS, Front, Beijing, China) and 1% penicillin/streptomycin solution (Sevenbio, Beijing, China). Cells were maintained in a humidified incubator at 37 °C under an atmosphere of 5% CO_2_. The medium was refreshed every 2–3 days, and cells were passaged at approximately 80–90% confluence using 0.25% trypsin-EDTA (Sevenbio, Beijing, China).

### 2.4. Cell Viability Assay

HepG2 cells were seeded into 96-well plates at a density of 5000 cells per well and cultured overnight. Then, they were treated with different doses of Eno or/and sorafenib for 24 or 48 h. After that, the medium was replaced with DMEM containing 10% CCK-8 reagent (Sevenbio, Beijing, China), and incubated at 37 °C for 30 min. Absorbance was measured at 450 nm using a microplate reader (Multiskan GO, Thermo, Waltham, MA, USA). Analysis of the effect of combination drugs: CCK-8 assay was used to detect the inhibition rate of Eno and sorafenib alone or in combination on HepG2 cells. The Q value was calculated using Jin’s formula [22] to evaluate whether the combined effect had a synergistic inhibitory influence on cell proliferation. Q = E_a+b_/(E_a_ + E_b_ − E_a_ × E_b_), where E_a_ and E_b_ are the inhibition rates of single drugs, respectively, and E_a+b_ is the inhibition rates of combination drugs. Q > 1.15 was synergistic effect, 0.85 ≤ Q ≤ 1.15 was additive effect, and Q < 0.85 was antagonistic effect. Cell viability (%) = (OD_treatment_ − OD_blank_)/(OD_control_ − OD_blank_) × 100%.

### 2.5. Colony Formation Assay

HepG2 cells were seeded into 6-well plates at a density of 1000 cells/well overnight, and then stimulated with Eno or/and sorafenib for 24 h. After that, the cells were cultured in complete medium for approximately 7–10 days, with the medium replenished every 3 days, until distinct macroscopic colonies became visible. Subsequently, the cells were gently washed twice with PBS, fixed with 4% paraformaldehyde for 30 min at room temperature, and then stained with 0.1% crystal violet solution for 20 min. The stained colonies were then photographed, and the number of colonies was quantified using ImageJ software (version 1.54g, Java 1.8.0_345, 64-bit).

### 2.6. Wound Healing Assay

HepG2 cells were cultured in 6-well plates until the density reached 95%. Then, the cells were scratched using a sterile 200 μL pipette tip and cultured for 24 h with Eno or/and sorafenib. Microscopic images at 100× magnification were captured at 0 and 24 h post-scratch. The area of cell wound healing was quantified using ImageJ software.

### 2.7. Migration and Invasion Assays

The abilities of cells to migrate and invade were assessed using Transwell inserts with an 8 μm pore diameter (Corning, NY, USA). In the invasion assay, the membrane was first covered with Matrigel (0.5 mg/mL; BD Biosciences, San Jose, CA, USA), while the migration assay was performed in the absence of any coating. Briefly, after stimulating HepG2 cells with Eno or/and sorafenib for 24 h, the cells were resuspended in serum-free DMEM and seeded into a 24-well transwell (upper chamber, pore size 8 μm, Corning) at a density of 2 × 10^5^ cells/well with 100 μL. The lower compartment was filled with 500 μL of DMEM supplemented with 10% FBS to act as a chemoattractant. After a 24 h incubation, non-migrated or non-invaded cells on the upper side of the membrane were gently wiped away with a cotton swab. The cells that had migrated through the membrane were then fixed with 4% paraformaldehyde, stained with 0.1% crystal violet, observed under an inverted microscope, and counted in five randomly selected fields per well using ImageJ software for quantification.

### 2.8. Iron Assay

HepG2 cells were seeded in culture dishes overnight and stimulated with Eno or/and sorafenib with or without liproxstatin-1 for another 24 h. After that, the cells were collected and subsequent operations were carried out according to the iron detection kit (Solarbio, Beijing, China) instructions. Briefly, each well received 500 μL of extraction solution, followed by ultrasonic disruption of cells on ice (set at 200 W power, with 3 s pulses and 7 s intervals, repeated 30 times). The samples were subsequently centrifuged at 4 °C for 10 min at 8000× *g*. The supernatant obtained was then uniformly combined with the appropriate detection reagent. After a 10 min incubation period at 25 °C to allow color development, absorbance was recorded at a wavelength of 510 nm. Fe^2+^ concentration was calculated according to the manufacturer’s formula.

### 2.9. Intracellular GSH Measurement

HepG2 cells were seeded in culture dishes overnight and stimulated with Eno or/and sorafenib with or without liproxstatin-1 for another 24 h. After that, the cells were collected and subsequent operations were carried out according to the GSH reagent kit (Solarbio, Beijing, China) instructions. Briefly, add 500 μL of extraction buffer to each well, followed by ultrasonic disruption on ice using a regimen of 3 s of sonication at 200 W power with a 10 s interval between pulses, repeated for 30 cycles. After sonication, centrifuge the lysates at 12,000× *g* for 10 min at 4 °C. Carefully collect the supernatant and mix it completely with the appropriate detection reagent. Allow the resulting mixture to incubate at room temperature for 2 min, then measure absorbance at 412 nm using a microplate spectrophotometer. Calculate the GSH concentration using the provided formula.

### 2.10. Intracellular LPO Measurement

HepG2 cells were seeded in culture dishes overnight and stimulated with Eno or/and sorafenib with or without liproxstatin-1 for another 24 h. After that, the cells were collected and subsequent operations were carried out according to the LPO content detection kit (Solarbio, Beijing, China) instructions. Briefly, cells were lysed in 500 µL extraction solution and ultrasonicated on ice (200 W, 3 s on, 7 s off, 30 cycles). Centrifuge the lysates at 8000× *g* for 10 min at 4 °C, then carefully collect the supernatant and mix thoroughly with the corresponding detection reagent. Incubate the mixture in a water bath at 100 °C for 60 min, followed by immediate cooling on ice. Centrifuge again at 8000× *g* for 10 min at room temperature. Measure the optical density of the resulting solution at 532 nm and 600 nm using a microplate reader. Calculate the LPO concentration according to the provided formula.

### 2.11. Quantitative Real-Time Polymerase Chain Reaction (qRT-PCR) Assay

Relative quantification of genes was performed by qRT-PCR. After treatment with Eno or/and sorafenib with or without liproxstatin-1 for 24 h, the cells were collected and subsequent operations were carried out according to the kit instructions. Briefly, total RNA was extracted using the Cell Fast RNA Extraction Kit (ABclonal, Wuhan, China). cDNA was synthesized with ABScript III RT Master Mix (ABclonal). qPCR was performed on a CFX96 Touch system (Bio-Rad, Hercules, CA, USA) using SYBR Green Master Mix (ABclonal). Gene expression levels were normalized to GAPDH. Primer sequences are listed in Table 1.

### 2.12. Western Blot Assay

HepG2 cells were seeded in culture dishes overnight and stimulated with Eno or/and sorafenib with or without liproxstatin-1 for another 24 h. After that, the cells were harvested and lysed, and the protein concentrations were determined using the BCA protein assay kit (KeyGen, Nanjing, China). Equal concentrations of protein were subjected to 12% SDS-PAGE gels and transferred to a PVDF membrane (Millipore, Burlington, MA, USA). Then, the membranes were blocked with freshly prepared 5% skim milk solution at room temperature for 1 h, and incubated with the primary antibody overnight at 4 °C. Subsequently, HRP-conjugated secondary antibodies were applied for 1 h at room temperature. Protein bands were then visualized and analyzed using a ChemiDoc gel imaging system (Bio-Rad, Hercules, CA, USA). As a loading control, β-actin was detected simultaneously. The relative protein expression level is determined by comparing the gray value of the target protein with that of the reference protein β-tubulin in each sample. The data values of the control group are normalized, and then the relative expression levels of the other treatment groups are calculated.

### 2.13. Animal Experiments

Twenty male C57BL/6 mice (5–6 weeks, 18–22 g) were obtained from the Laboratory Animal Center of Ningxia Medical University. Mice were randomly divided into four groups of five mice per group according to body weight and housed in standardized SPF barrier facilities with temperature (22 ± 1 °C), light (12–12 h light–dark cycles), relative humidity (50 ± 5%) and food and water freely. Animal experiments were strictly conducted in accordance with the “Guide for the Care and Use of Laboratory Animals” and strictly followed the ethical standards approved by the Institutional Animal Care and Use Committee of Ningxia Medical University (IACUC—2025094).

After 3 days of adaptive feeding, 100 µL of 2 × 10^6^ Hepal-6 cells suspension was injected subcutaneously into the right armpit of each mouse to establish subcutaneous transplanted tumors. Mice body weight and tumor volume were monitored regularly. When the tumor volume reached approximately 60 mm^3^, drug intervention was performed at 0.1 mL/10 g per mouse: The control group (normal saline), Eno group (50 mg/kg/d, gavage), sorafenib group (20 mg/kg/d, intraperitoneal injection), and combination group (Eno 50 mg/kg/d + sorafenib 20 mg/kg/d). At the same time, the body weight of mice was weighed every day, and the tumor volume of mice was measured every 3 days. On the 9th day after inoculation, all mice were humanely euthanized using a CO_2_ inhalation system (SMQ-II-Q, Shanghai, China), and tumor tissues were excised, weighed, fixed in 4% paraformaldehyde, and stored in 70% ethanol for subsequent paraffin embedding.

### 2.14. Hematoxylin–Eosin (H&E) Staining

Paraffin embedded tumor tissue sections were deparaffinized and hydrated following the manufacturer’s instructions of the immunohistochemical kit (ZSGB-BIO, Beijing, China). After 5 min of hematoxylin staining, nuclei were kept blue and then differentiated in 1% ethanol hydrochloride for several seconds, and the cytoplasm was stained with eosin for 1 min. Subsequently, sections were dehydrated with gradient alcohol, made transparent with xylene, and sealed with neutral resin. Under a 200× upright microscope (E200, Nikon, Tokyo, Japan), five representative fields of view were randomly selected for histopathological assessment. The histological evaluation mainly focused on the morphology of tumor cells, the area of necrotic regions, and the ratio of tumor to stroma.

### 2.15. Immunofluorescence Assays

For detailed procedures, refer to the method of Zaqout et al. [23]. In brief, the tissue sections were first dewaxed, then fixed with 4% paraformaldehyde at room temperature for 30 min, followed by permeabilization with 0.1% Triton X-100 (Sevenbio, Beijing, China) for 10 min, and finally blocked with 5% BSA (Sevenbio, Beijing, China) at room temperature for 1 h. After blocking, the sections were incubated with the primary antibody overnight at 4 °C. Sections were then incubated with FITC-conjugated secondary antibodies for 50 min at room temperature, counterstained with DAPI (Sevenbio, Beijing, China) for 10 min in the dark, and imaged using a fluorescence microscope (N1-U, DS-R12, Nikon, Tokyo, Japan).

### 2.16. Qualitative and Quantitative Analysis of the Main Active Components of Eno by UPLC-MS/MS

The composition of Eno was determined in the positive ion mode. The Eno powder was dissolved in methanol/water (50:50, *v*/*v*) to prepare the Eno stock solution. The samples were separated using a Waters ACQUITY UPLC HSS T3 (2.1 × 100 mm, 1.8 μm) column and analyzed by a high-performance liquid chromatography (1290 Infinity II, Agilent, Santa Clara, CA, USA) coupled with a triple quadrupole mass spectrometer (Qtrap 6500, AB Sciex, Framingham, MA, USA). Quantification was performed using the external standard method.

### 2.17. Statistical Analysis

Statistical analysis of the experimental data was conducted using GraphPad Prism 8.4.3 software, and was presented as mean ± standard deviation (SD). The comparison of the two sets of data was conducted using the unpaired two-tailed Student’s *t*-test. For the comparison of multiple sets of data, the one-way analysis of variance (ANOVA) was employed. *p* < 0.05 was regarded as statistically significant.

## 3. Results

### 3.1. Eno Inhibits the Viability and Proliferation of HepG2 Cells

**Figure 1:**
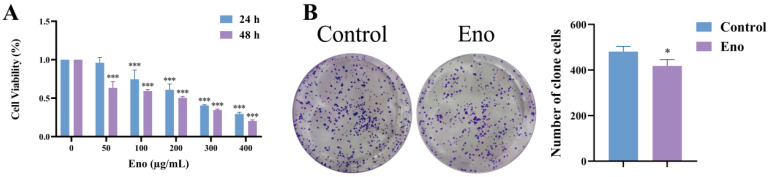
Eno inhibits the viability and proliferation of HepG2 cells. (**A**) Viability of HepG2 cells treated with Eno for 24 or 48 h, as determined by CCK-8 assay. (**B**) Colony formation ability of HepG2 cells following Eno (100 μg/mL) treatment for one week. *n* = 3 biologically independent samples. * is the comparison between the experimental group and the control group, * *p* < 0.05 and *** *p* < 0.001. *p* values were determined by unpaired two-tailed Student’s *t*-tests.

We first evaluated the impact of Eno on HepG2 cell viability and proliferative capacity. As depicted in Figure 1A, CCK-8 assays demonstrated that treatment with Eno (50, 100, 200, 300 and 400 μg/mL) for 24 h and 48 h led to a dose- and time-dependent suppression of cell viability. A significant reduction in viability was observed at concentrations of 100 μg/mL and above following 24 h of exposure. Consequently, a 24 h treatment with 100 μg/mL Eno was selected for subsequent experiments. The colony formation assay further confirmed that Eno markedly suppressed the proliferative capacity of HepG2 cells (Figure 1B). Collectively, these findings indicate that Eno effectively inhibits both the viability and proliferation of HepG2 cells.

### 3.2. Eno Inhibits the Migration and Invasion of HepG2 Cells

**Figure 2:**
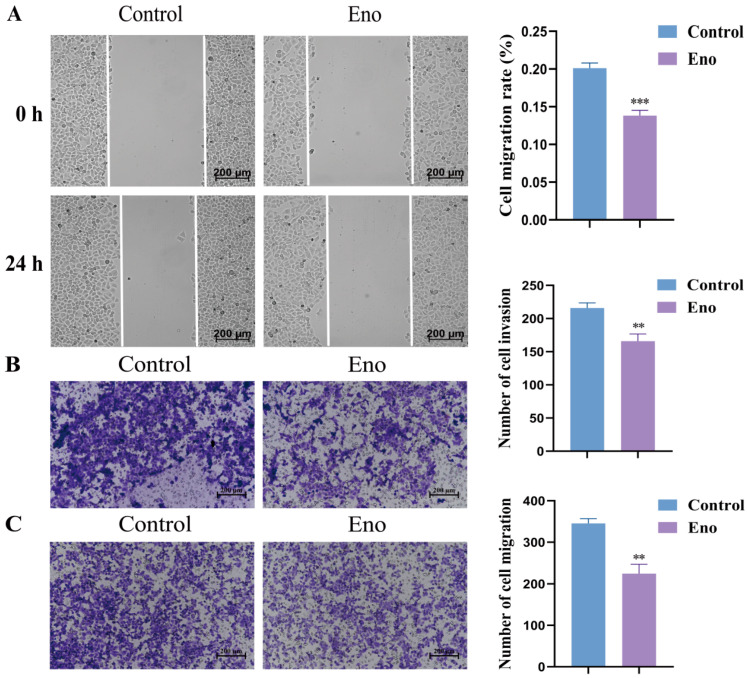
Eno inhibits the migration and invasion of HepG2 cells. (**A**) Representative images (100×) of the wound healing assay demonstrating the inhibitory effect of Eno (100 μg/mL) on the lateral migration of HepG2 cells. (**B**,**C**) Representative images (100×) of transwell matrigel invasion assay (**B**) and migration assay (**C**), confirmed that Eno (100 μg/mL) treatment for 24 h inhibited the longitudinal migration and invasion ability of HepG2 cells. *n* = 3 biologically independent samples. * is the comparison between the experimental group and the control group, ** *p* < 0.01 and *** *p* < 0.001. *p* values were determined by unpaired two-tailed Student’s *t*-tests.

We next assessed the effect of Eno on the metastatic potential of HepG2 cells. Scratch wound healing assays showed that treatment with Eno for 24 h significantly reduced wound closure, indicating impaired the cell migratory capacity (Figure 2A). Consistent with this finding, transwell assays demonstrated that Eno markedly decreased the number of migrating (Figure 2B) and invading (Figure 2C) HepG2 cells after 24 h of treatment. Taken together, these results demonstrate that Eno effectively suppresses both the migratory and invasive capabilities of HepG2 cells.

### 3.3. Eno Inhibits HepG2 Cell Function Through Ferroptosis

**Figure 3:**
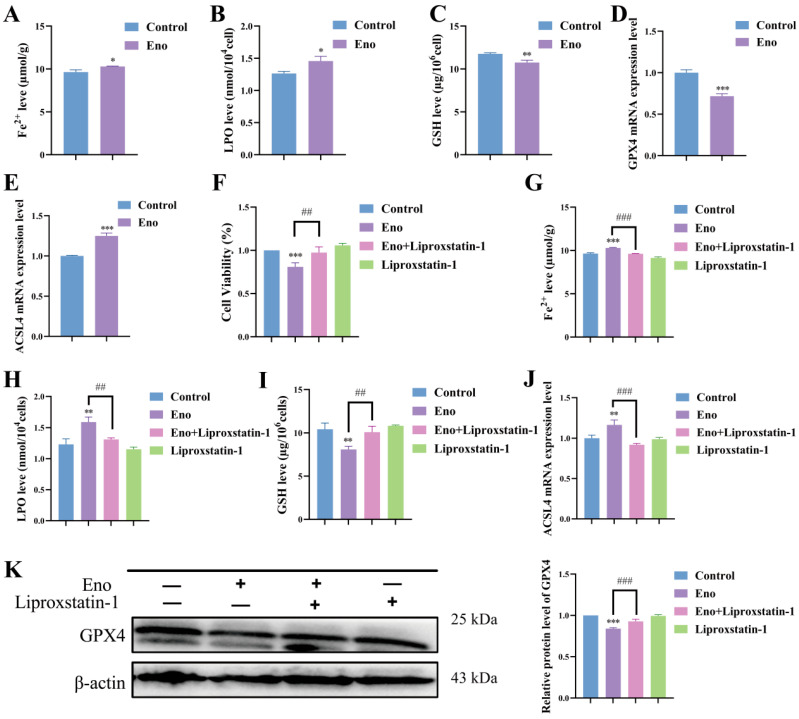
Eno inhibits HepG2 cell function through ferroptosis. (**A**–**C**) Intracellular Fe^2+^ levels (**A**), LPO content (**B**), and GSH levels (**C**) in HepG2 cells after Eno (100 μg/mL) treatment for 24 h, as determined by corresponding assay kits. (**D**,**E**) mRNA expression levels of ferroptosis-related genes (GPX4, ACSL4) in Eno-treated (100 μg/mL) HepG2 cells for 24 h, measured by qRT-PCR. (**F**) Viability of HepG2 cells treated with Eno (100 μg/mL) and/or Liproxstatin-1 (1 μM) for 24 h, assessed by CCK-8 assay. (**G**–**I**) Changes in intracellular Fe^2+^ (**G**), LPO (**H**), and GSH (**I**) levels in HepG2 cells following treatment with Eno (100 μg/mL) and/or Liproxstatin-1 (1 μM) for 24 h, measured using corresponding assay kits. (**J**) mRNA expression of ferroptosis-related genes ACSL4 in HepG2 cells treated with Eno (100 μg/mL) and/or Liproxstatin-1 (1 μM) for 24 h, analyzed by qRT-PCR. (**K**) GPX4 protein expression in HepG2 cells after treatment with Eno (100 μg/mL) and/or Liproxstatin-1 (1 μM) for 24 h, detected by Western blotting. *n* = 3 biologically independent samples. * is the comparison between the experimental group and the control group, and # is the comparison between the groups, * *p* < 0.05, ** *p* < 0.01, *** *p* < 0.001 and ^##^ *p* < 0.01, ^###^ *p* < 0.001. *p* values were determined by unpaired two-tailed Student’s *t*-tests or one-way ANOVA.

To explore the mode of cell death induced by Eno, we analyzed key biomarkers associated with ferroptosis. As illustrated in Figure 3A–C, intracellular Fe^2+^ and LPO levels were significantly elevated in Eno-treated HepG2 cells, whereas GSH content was substantially depleted. At the gene level, Eno treatment resulted in a down-regulation of GPX4 mRNA expression and a concurrent up-regulation of ACSL4 mRNA expression (Figure 3D,E). To further validate the involvement of ferroptosis, we employed Liproxstatin-1, a specific inhibitor of ferroptosis. Pre-treatment with Liproxstatin-1 significantly rescued Eno-induced cytotoxicity (Figure 3F) and effectively reversed the Eno-mediated alterations in Fe^2+^, LPO, GSH levels, as well as ACSL4 and GPX4 expression (Figure 3G–K). These findings strongly suggest that the anti-HCC activity of Eno is primarily mediated through the induction of ferroptosis.

### 3.4. Eno-Induced Ferroptosis Is Associated with Suppression of the p62/Keap1/Nrf2/HO-1 Signaling Pathway in HepG2 Cells

**Figure 4:**
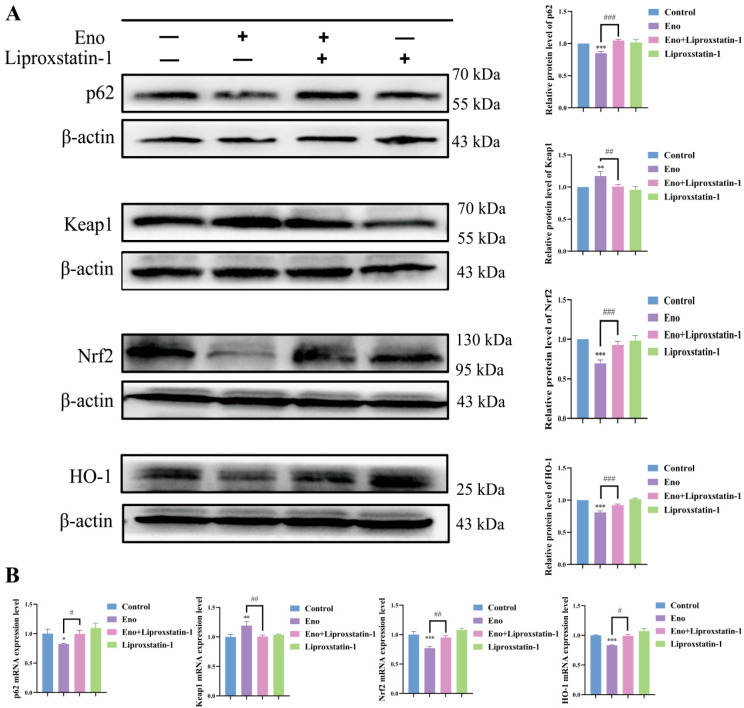
Eno-induced ferroptosis is associated with suppression of the p62/Keap1/Nrf2/HO-1 signaling pathway in HepG2 cells. HepG2 cells were treated with Eno (100 μg/mL) and/or liprostatin-1 (1 μM) for 24 h, and then the cells were collected for detection. (**A**) The protein expression levels of p62, Keap1, Nrf2 and HO-1 in HepG2 cells were evaluated by Western blot analysis. (**B**) mRNA expression levels of corresponding genes were determined by qRT-PCR. *n* = 3 biologically independent samples. * is the comparison between the experimental group and the control group, and # is the comparison between the groups, * *p* < 0.05, ** *p* < 0.01, *** *p* < 0.001 and ^#^ *p* < 0.05, ^##^ *p* < 0.01, ^###^ *p* < 0.001. *p* values were determined by unpaired two-tailed Student’s *t*-tests or one-way ANOVA.

Recent studies have demonstrated that Nrf2 acts as a negative regulator of ferroptosis, primarily through its modulation by the substrate adaptor protein p62 via interaction with Keap1 [24]. HO-1, a downstream effector of Nrf2, promotes ferroptosis by facilitating iron release, which contributes to the accumulation of lipid peroxides [25]. To explore the potential involvement of the p62/Keap1/Nrf2/HO-1 signaling pathway in Eno-induced ferroptosis, we assessed the expression alterations within this pathway through qRT-PCR and Western blotting. As demonstrated in Figure 4, treatment with Eno markedly reduced the expression of p62, Nrf2, and its downstream target HO-1, while enhancing Keap1 expression at both protein and mRNA levels. Co-treatment with Liproxstatin-1 effectively reversed these Eno-induced changes, restoring the expression levels of p62, Nrf2, HO-1, and Keap1 towards baseline. These results indicate that Eno induces ferroptosis in HepG2 cells is correlated with impaired function of the p62/Keap1/Nrf2/HO-1 signaling axis.

### 3.5. Eno and Sorafenib Exhibit Synergistic Cytotoxicity to HepG2 Cells

**Figure 5:**
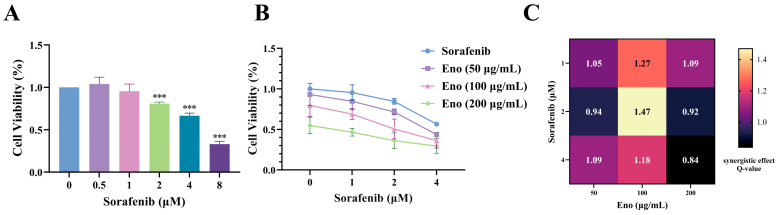
Eno and sorafenib exhibit synergistic cytotoxicity to HepG2 cells. (**A**) Cell viability after 24 h of sorafenib treatment was assessed by the CCK-8 assay. (**B**) Effects of the combined treatment for 24 h of sorafenib and Eno on cell viability were measured by the CCK-8 assay. (**C**) The Q-value method was used to evaluate the synergistic effect for 24 h of the two drugs, and the results are presented as matrix plots. Q > 1.15 was synergistic effect, 0.85 ≤ Q ≤ 1.15 was additive effect, and Q < 0.85 was antagonistic effect. *n* = 3 biologically independent samples. * is the comparison between the experimental group and the control group, *** *p* < 0.001. *p* values were determined by unpaired two-tailed Student’s *t*-tests.

To further investigate the potential synergistic effect of Eno in enhancing sorafenib’s anti-HCC activity, we first assessed the impact of sorafenib, either alone or combined with Eno, on HepG2 cell viability. The results showed that sorafenib monotherapy inhibited HepG2 cell viability in a dose-dependent manner (Figure 5A). Subsequently, we used Jin’s formula to calculate Q values to initially assess the synergistic effect of Eno and sorafenib. When the Q value was greater than 1.15, it was defined as a synergistic effect. Our results showed that when Eno was at 100 μg/mL and sorafenib was at 2 μM, the strongest synergistic effect was observed (Q = 1.47) (Figure 5C). This optimal synergistic combination was therefore used for subsequent combination therapy studies.

### 3.6. Eno Synergistically Enhances the Inhibitory Effect of Sorafenib on the Proliferation and Metastasis of HepG2 Cells

**Figure 6:**
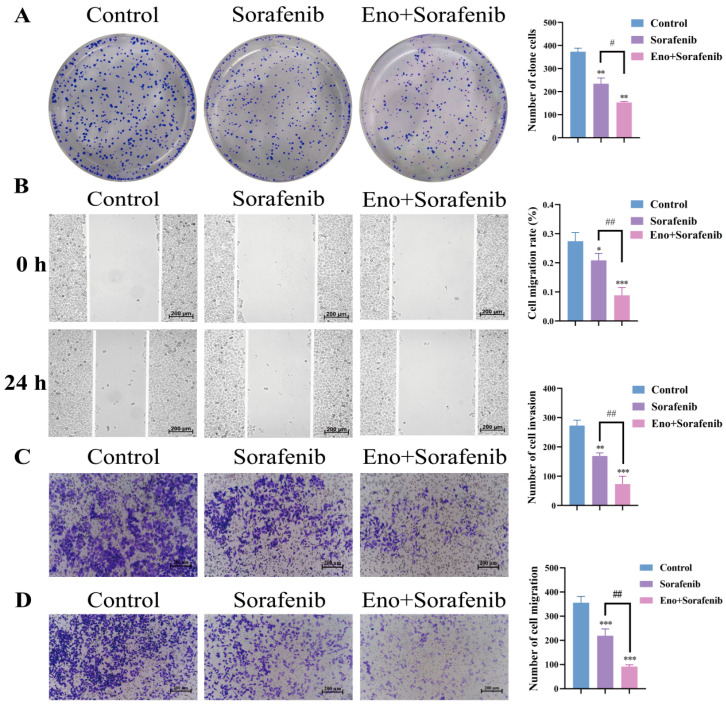
Eno synergistically enhances the inhibitory effect of sorafenib on the proliferation and metastasis of HepG2 cells. (**A**) Clonogenic assay of HepG2 cells treated with sorafenib (2 μM) alone or in combination with Eno (100 μg/mL) for one week. (**B**) Cell migration assessed by wound healing assay in HepG2 cells treated with sorafenib (2 μM) alone or in combination with Eno (100 μg/mL) (100×). (**C**,**D**) Transwell invasion (**C**) and migration (**D**) assays of HepG2 cells following treatment for 24 h with sorafenib (2 μM) alone or in combination with Eno (100 μg/mL) (100×). *n* = 3 biologically independent samples. * is the comparison between the experimental group and the control group, and # is the comparison between the groups, * *p* < 0.05, ** *p* < 0.01, *** *p* < 0.001 and ^#^ *p* < 0.05, ^##^ *p* < 0.01. *p* values were determined by unpaired two-tailed Student’s *t*-tests or one-way ANOVA.

The synergistic effects on malignant phenotypes were further investigated. The combination therapy resulted in a greater inhibition of colony formation compared to sorafenib alone (Figure 6A). Wound healing, transwell migration, and matrigel invasion assays consistently demonstrated that the Eno–sorafenib combination was significantly more effective in suppressing cell migration and invasion than sorafenib monotherapy, as shown in Figure 6B–D. These results indicate that Eno significantly enhances sorafenib’s ability to suppress both the growth and metastatic potential of HCC cells in a synergistic manner.

### 3.7. Eno Synergistically Promotes Ferroptosis in HepG2 Cells with Sorafenib

**Figure 7:**
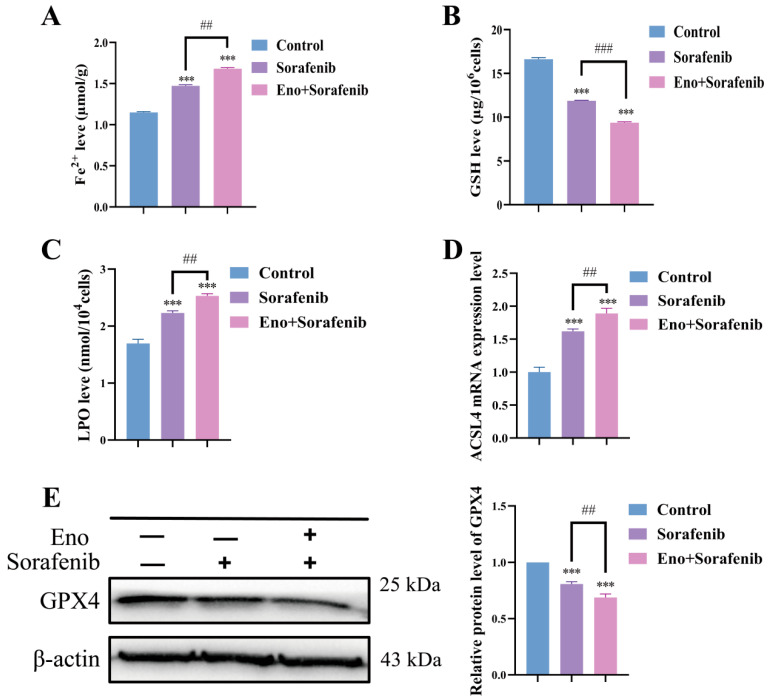
Eno synergistically promotes ferroptosis in HepG2 cells with sorafenib. (**A**–**C**) Effects of sorafenib (2 μM) alone or in combination for 24 h with Eno (100 μg/mL) on intracellular Fe^2+^ levels (**A**), GSH levels (**B**) and LPO content (**C**) in HepG2 cells, as measured by corresponding commercial assay kits. (**D**) mRNA expression levels of ferroptosis-related genes ACSL4 in HepG2 cells treated for 24 h with sorafenib (2 μM) alone or combined with Eno (100 μg/mL), as determined by qRT-PCR. (**E**) GPX4 protein expression in HepG2 cells following treatment for 24 h with sorafenib (2 μM) alone or in combination with Eno (100 μg/mL), detected by Western blotting analysis. *n* = 3 biologically independent samples. * is the comparison between the experimental group and the control group, and # is the comparison between the groups, *** *p* < 0.001 and ^##^ *p* < 0.01, ^###^ *p* < 0.001. *p* values were determined by unpaired two-tailed Student’s *t*-tests or one-way ANOVA.

Sorafenib has been well-established as an inducer of ferroptosis [26], and our preliminary experiments further demonstrate that Eno alone can trigger ferroptosis. To investigate the potential synergistic interaction between Eno and sorafenib in promoting ferroptosis in HepG2 cells, we assessed a panel of key biochemical and molecular markers associated with this process. As shown in Figure 7, sorafenib effectively induced ferroptosis in HepG2 cells, consistent with previous reports. The combination therapy led to a more pronounced accumulation of intracellular Fe^2+^ and LPO, alongside a more severe depletion of GSH, compared to sorafenib alone. Furthermore, the combination treatment resulted in a higher induction of ACSL4 mRNA and a more profound suppression of GPX4 protein expression than sorafenib alone. These data indicate that Eno synergistically enhances sorafenib-induced ferroptosis.

### 3.8. Eno Enhances Sorafenib-Induced Ferroptosis Is Accompanied by a Synergistic Inhibition of the p62/Keap1/Nrf2/HO-1 Signaling Pathway in HepG2 Cells

**Figure 8:**
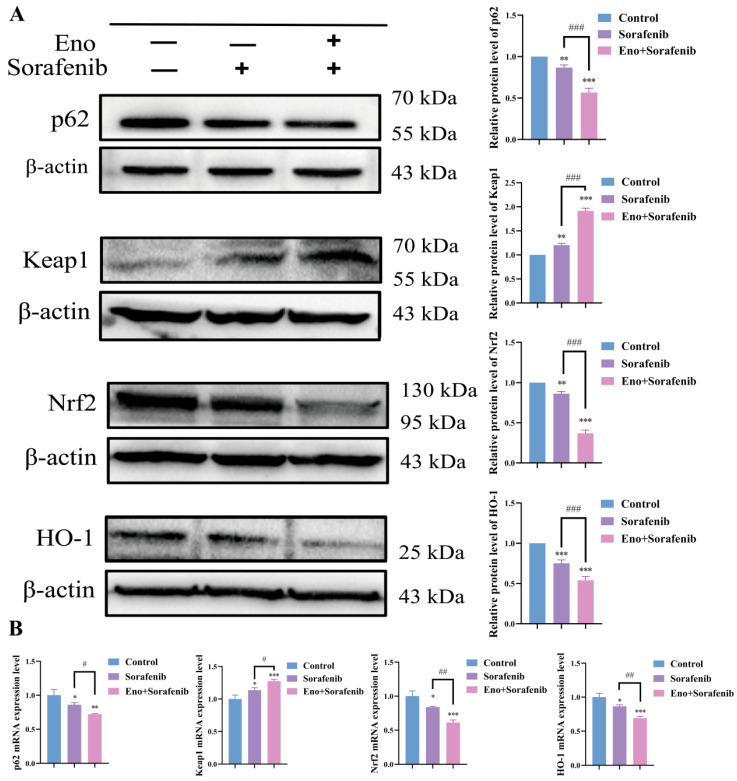
Eno enhances sorafenib-induced ferroptosis is accompanied by a synergistic inhibition of the p62/Keap1/Nrf2/HO-1 signaling pathway in HepG2 cells. HepG2 cells were treated with sorafenib (2 μM) or sorafenib (2 μM) in combination with Eno (100 μg/mL) for 24 h, cells were harvested for assay. (**A**) The protein expression levels of p62, Keap1, Nrf2 and HO-1 in HepG2 cells were evaluated by Western blot analysis. (**B**) mRNA expression levels of corresponding genes were determined by qRT-PCR. *n* = 3 biologically independent samples. * is the comparison between the experimental group and the control group, and # is the comparison between the groups, * *p* < 0.05, ** *p* < 0.01, *** *p* < 0.001 and ^#^ *p* < 0.05, ^##^ *p* < 0.01, ^###^ *p* < 0.001. *p* values were determined by unpaired two-tailed Student’s *t*-tests or one-way ANOVA.

To gain deeper insights into the molecular mechanisms by which Eno and sorafenib cooperatively induce ferroptosis in HepG2 cells, we examined the expression changes in the p62/Keap1/Nrf2/HO-1 signaling pathway at both mRNA and protein levels. Our results indicated that the combination of Eno and sorafenib caused a more substantial down-regulation of p62, Nrf2, and HO-1 expression, and a more pronounced up-regulation of Keap1, compared to sorafenib monotherapy, at both the protein and transcript levels (Figure 8A,B). This suggests that the synergistic induction of ferroptosis is accompanied by an enhanced suppression of the p62/Keap1/Nrf2/HO-1 signaling axis.

### 3.9. Eno Combined with Sorafenib Synergistically Inhibits the Growth of Subcutaneous HCC Xenografts in Mice

**Figure 9:**
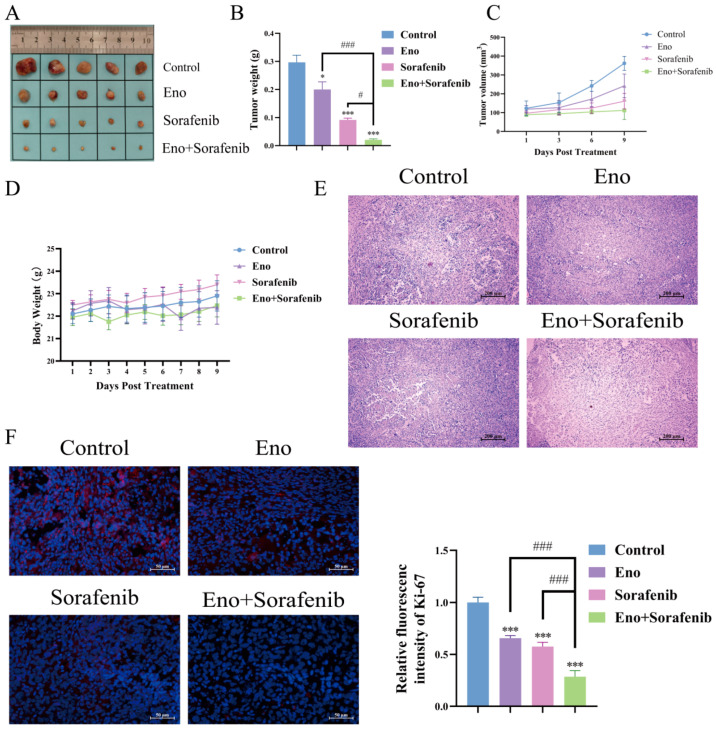
Eno combined with sorafenib synergistically inhibits the growth of subcutaneous HCC xenografts in mice. (**A**) Anatomical map of the mouse tumor. (**B**) Statistical plot of tumor weight. (**C**) Statistical plot of tumor volume. (**D**) Mouse weight statistics chart. (**E**) Morphological assessment of tumor tissues by H&E staining following treatment with Eno, sorafenib, or their combination (200×). (**F**) Evaluation of tumor cell proliferation by immunofluorescence staining of the Ki-67 marker (400×). *n* = 5 biologically independent samples. * is the comparison between the experimental group and the control group, and # is the comparison between the groups, * *p* < 0.05, *** *p* < 0.001 and ^#^ *p* < 0.05, ^###^ *p* < 0.001. *p* values were determined by unpaired two-tailed Student’s *t*-tests or one-way ANOVA.

Given the synergistic inhibitory effect of Eno and sorafenib on HCC cells observed in vitro, we established a xenograft model by subcutaneously inoculating Hepa1-6 cells into C57BL/6 mice to further assess the individual and combined effects on HCC tumor growth. Our results demonstrated that both Eno and sorafenib as single agents significantly suppressed tumor growth (the inhibition rates reached 33% and 55% respectively). However, the combination therapy produced the most potent anti-tumor effect (the inhibition rates reached 70%), as evidenced by significantly smaller tumor size (Figure 9A), reduced tumor weight (Figure 9B), and suppressed tumor volume progression (Figure 9C) compared to all other groups. No notable changes in body weight were observed across the treatment groups (Figure 9D), indicating limited systemic toxicity. Histopathological examination by H&E staining revealed extensive necrosis in the combination group (Figure 9E). Furthermore, immunofluorescence analysis for the proliferation marker Ki-67 showed the most substantial reduction in proliferating cells within the tumors from the combination treatment group (Figure 9F). Collectively, these findings indicate that both Eno and sorafenib can inhibit HCC tumor growth in vivo, and their combination exerts a synergistic antitumor effect.

### 3.10. Eno Combined with Sorafenib Synergistically Inhibits the Progression of HCC Which Is Related to the Suppression of the p62/Keap1/Nrf2/HO-1 Signaling Pathway In Vivo

**Figure 10:**
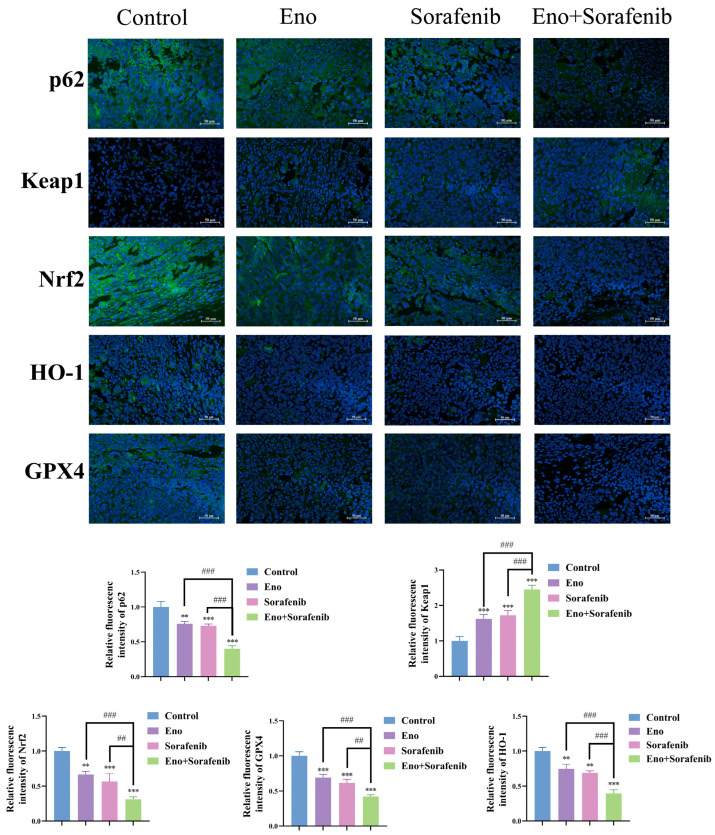
Eno combined with sorafenib synergistically inhibits the progression of HCC which is related to the suppression of the p62/Keap1/Nrf2/HO-1 signaling pathway in vivo. Scale bar: 50 μm. Representative immunofluorescence images (400×) showing the expression of p62, Keap1, Nrf2, HO-1, and GPX4 in tumor tissues following treatment with Eno, sorafenib, or their combination. *n* = 5 biologically independent samples. * is the comparison between the experimental group and the control group, and # is the comparison between the groups, ** *p* < 0.01, *** *p* < 0.001 and ^##^ *p* < 0.01, ^###^ *p* < 0.001. *p* values were determined by unpaired two-tailed Student’s *t*-tests or one-way ANOVA.

To further insight into the molecular mechanisms of the synergistic inhibition of HCC tumor growth by Eno and sorafenib in vivo, we examined expression changes in the ferroptosis marker GPX4 and key components of the p62/Keap1/Nrf2/HO-1 signaling pathway using immunofluorescence analysis of tumor tissues. Our findings showed that the expression of p62, Nrf2, HO-1, and GPX4 was most significantly down-regulated, while Keap1 expression was most markedly up-regulated, in the combination treatment group compared to monotherapies or control (Figure 10). These in vivo results confirm that the synergistic antitumor effect of Eno and sorafenib is associated with the enhancement of ferroptosis and the cooperative inhibition of the p62/Keap1/Nrf2/HO-1 signaling pathway.

## 4. Discussion

**Figure 11:**
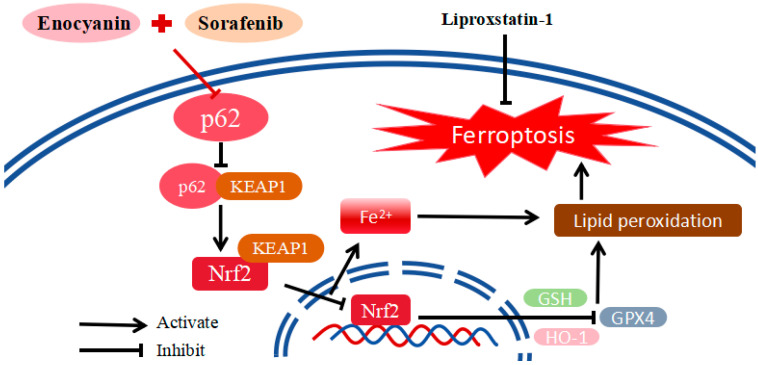
Proposed mechanism of ferroptosis induction by Eno in combination with sorafenib in HCC cells. The schematic diagram illustrates our model wherein the anti-tumor effects of the Eno/sorafenib combination are associated with the modulation of the p62/Keap1/Nrf2/HO-1 pathway. The combination treatment down-regulates p62, leading to the release of Keap1. The liberated Keap1 then binds and sequesters Nrf2 in the cytoplasm, thereby inhibiting its nuclear translocation. This results in the down-regulation of key antioxidant proteins, including HO-1, and a reduction in cellular antioxidant capacity (e.g., decreased levels of GSH and GPX4). These changes contribute to the accumulation of Fe^2+^ and lipid peroxides (LPO), ultimately triggering ferroptosis.

The clinical treatment of HCC still faces many bottlenecks, including multidrug resistance and insufficient therapeutic sensitivity, which seriously hinder the improvement of patient prognosis [27,28]. Sorafenib, a first-line treatment for advanced HCC, often exhibits transient efficacy due to acquired resistance, underscoring the need for innovative strategies to resensitize tumors [11]. Our study provides compelling evidence that Eno, a natural anthocyanin-rich extract from grapes, not only exhibits intrinsic anti-HCC activity, but also acts synergistically with sorafenib to overcome treatment resistance. Mechanistically, these effects are associated with the suppression of the p62/Keap1/Nrf2/HO-1 signaling pathway and the cooperative induction of ferroptosis, as illustrated in Figure 11.

Natural products offer unique advantages in cancer therapy, such as wide-ranging sources, multi-target capabilities, low toxicity, and diverse biological activities [29,30]. Anthocyanins, as polyphenolic compounds extensively present in plants, have demonstrated significant anti-proliferative activity in a variety of tumor cells, such as A549 lung cancer cells and Hela cervical cancer cells [31,32]. The main component of Eno is anthocyanin. In this study, six active components were identified in Eno by UPLC-MS/MS, namely cyanidin-3-O-glucoside, peonidin-3-O-glucoside, delphinidin-3,5-O-diglucoside chloride, delphinidin-3-O-rutinoside chloride, malvidin-3,5-O-diglucoside chloride, and pelargonidin-3-O-glucoside. Among them, cyanidin-3-O-glucoside has the highest content, reaching 9026.22 μg/g (Table S1). This multi-component characteristic may endow Eno with a pleiotropic ability to modulate cell death pathways, which is different from traditional single-target drugs. Therefore, subsequent research will focus on the most abundant cyanidin-3-O-glucoside to explore in depth whether it is the key active component for the biological functions of Eno. Previous studies have shown that Eno has been proven to have biological functions such as inhibiting acid phosphatase activity and anti-inflammation [21]. This study further broadens the understanding of the biological functions of Eno and confirms for the first time that Eno suppresses HepG2 cell proliferation and migration by inducing ferroptosis, as evidenced by elevated Fe^2+^ and LPO levels, GSH depletion, and ACSL4 upregulation alongside GPX4 downregulation (Figure 1, Figure 2 and Figure 3). The ferroptosis dependence was further confirmed using Liproxstatin-1, which reversed Eno-induced cytotoxicity (Figure 3). These findings highlight Eno’s potential as a ferroptosis-based therapeutic agent.

Ferroptosis, a form of regulatory cell death, characterized by intracellular iron, ACSL4 and LPO accumulation, glutathione depletion, and reduced GPX4 activity [33,34]. Ferroptosis is regarded as one of the key mechanisms of sorafenib treatment response [35]. However, tumor cells often activate compensatory antioxidant pathways, leading to treatment failure [36]. Recent studies have shown that the combination of natural products with chemotherapeutic drugs or targeted drugs can effectively enhance the sensitivity of tumor cells to drugs, thereby improving the treatment outcomes [37]. For example, ginsenoside Rg3 and dihydroartemisinin have been proven to synergize with sorafenib by inducing ferroptosis or inhibiting energy metabolism [38,39]. Our data reveal that the combined treatment of Eno and sorafenib exhibits a strong synergistic effect in inhibiting the viability, proliferation, migration, and invasion of HepG2 cells (Figure 5 and Figure 6), and can significantly enhance sorafenib’s ferroptosis-inducing effects, resulting in amplified Fe^2+^ and LPO accumulation, GSH depletion, and ACSL4, GPX4 dysregulation (Figure 7). This combined effect may demonstrate the potential of Eno as a ferroptosis inducer to overcome sorafenib resistance.

A pivotal finding of our study concerns the sophisticated regulation of the p62/Keap1/Nrf2 signaling node. Abnormal activation of Nrf2 downstream target genes in this pathway, such as NAD(P)H: quinone oxidoreductase 1 (NQO1), HO-1, and ferritin heavy chain 1 (FTH1), inhibits ferroptosis by enhancing the antioxidant capacity of cells, leading to the resistance of tumor cells to sorafenib [15,40]. Our study (Figure 4 and Figure 8) demonstrates that Eno alone or combined with sorafenib down-regulates p62, releasing Keap1 to facilitate Nrf2 degradation, thereby inhibiting the Nrf2-HO-1 axis and creating a permissive environment for lipid peroxidation, which significantly lowers the threshold for induction of ferroptosis. In vivo, the combination therapy robustly inhibited tumor growth in a xenograft model, with enhanced suppression of p62, Nrf2, HO-1, and GPX4, and increased Keap1 (Figure 9 and Figure 10), further validating the role of this pathway.

In summary, this study demonstrates that the combination of Eno and sorafenib synergistically induces ferroptosis in HCC, significantly inhibits tumor growth, and does not induce obvious systemic toxicity in the tested models. These findings provide a preliminary experimental basis for developing natural product-based combination strategies against liver cancer and suggest the potential of Eno in modulating sorafenib sensitivity, laying a foundation for its further investigation. However, this study has several limitations. First, the precise molecular targets responsible for the observed synergy between Eno and sorafenib remain to be conclusively identified. Second, the therapeutic efficacy has not been fully validated across a broader range of liver cancer models (e.g., patient-derived xenografts, models of different etiologies). Third, the in vivo pharmacokinetic profile and biodistribution of Eno, both alone and in combination, require systematic analysis. Finally, the specific bioactive component(s) within Eno and their structure–activity relationships are yet to be elucidated. Future research should therefore focus on: (1) employing high-throughput screening and AI-assisted target prediction, coupled with CRISPR-Cas9 gene editing validation, to precisely identify the core targets through which the Eno–sorafenib combination regulates ferroptosis; (2) expanding efficacy evaluations in more clinically relevant models; (3) conducting comprehensive pharmacokinetic and pharmacodynamic studies; and (4) isolating and characterizing the active constituents of Eno.

In conclusion, Eno, as a naturally derived agent with features such as wide availability and a favorable safety profile in our study, shows promise in the field of cancer combination therapy. It may hold potential as a candidate agent for enhancing the efficacy of sorafenib, warranting further translational development.

## Figures and Tables

**Table 1:** Primers used in this study.

Gene	Forward Primer	Reverse Primer
Human p62	AATCAGCTTCTGGTCCATCG	TTCTTTTCCCTCCGTGCTC
Human Keap1	AACAGAGACGTGGACTTTCG	GTGTCTGTATCTGGGTCGTAAC
Human Nrf2	CAAAAGGAGCAAGAGAAAGCC	TCTGATTTGGGAATGTGGGC
Human HO-1	TCAGGCAGAGGGTGATAGAAG	TTGGTGTCATGGGTCAGC
Human GPX4	CGCTGTGGAAGTGGATGAAG	TTGTCGATGAGGAACTGTGG
Human ACSL4	CCAAAGAACACCATTGCCATC	AGCCTCAGATTCATTTAGCCC
Human GAPDH	ACATCGCTCAGACACCATG	TGTAGTTGAGGTCAATGAAGGG

## Data Availability

All the original contributions of this research have been included in this article. If you have any further questions, you can contact the corresponding authors.

## Supplementary Materials

The following supporting information can be downloaded at: https://www.mdpi.com/article/10.3390/cimb48040357/s1.

## Abbreviations

The following abbreviations are used in this manuscript:

HCC	Hepatocellular carcinoma
Eno	Enocyanin
LPO	Lipid Peroxidation
ACSL4	Acyl-CoA Synthetase Long Chain Family Member 4
GSH	Glutathione
GPX4	Glutathione Peroxidase 4
p62	Sequestosome 1
Keap1	Kelch-like ECH-associated protein 1
Nrf2	Nuclear factor erythroid 2-related factor 2
HO-1	Heme Oxygenase-1
GAPDH	Glyceraldehyde-3-Phosphate Dehydrogenase
VEGFR	Vascular Endothelial Growth Factor Receptor
PDGFR	Platelet-Derived Growth Factor Receptor
SF	Sorafenib
NAD(P)H	Nicotinamide Adenine Dinucleotide (Phosphate)
NQO1	NAD(P)H: Quinone Dehydrogenase 1
FTH1	Ferritin Heavy Chain 1
