# Supplementary material for: Enocyanin Synergistically Enhances Sorafenib Sensitivity in Hepatocellular Carcinoma via Ferroptosis Induction Associated with p62/Keap1/Nrf2/HO-1 Pathway Inhibition
Source: Curr Issues Mol Biol. 2026 Mar 28;48(4):357. doi: 10.3390/cimb48040357 (PMC13114673; doi:10.3390/cimb48040357)
Supplement: Supplementary file 1 [file cimb-48-00357-s001.zip › cimb-4181045-supplementary.pdf]

Supplementary Materials

Enocyanin synergistically enhances sorafenib sensitivity in hepatocellular carcinoma via ferroptosis induction associated with p62/Keap1/Nrf2/HO-1 pathway inhibition

Mengting Tian [1,†], Jing Ma [2,†], Tingting Wei [3], Kunqi Meng [4], Yingmeng Xia [2], Xue Zong [4], Changcai Bai [1,*] and Zhisheng Wang [2,*]

[1] School of Pharmacy, Ningxia Medical University, Yinchuan, Ningxia Hui Autonomous Region, 750004, P.R. China

[2] School of Inspection, Ningxia Medical University, Yinchuan, Ningxia Hui Autonomous Region, 750004, P.R. China

[3] The First Clinical College, Ningxia Medical University, Yinchuan, Ningxia Hui Autonomous Region, 750004, P.R. China

[4] School of Public Health, Ningxia Medical University, Yinchuan, Ningxia Hui Autonomous Region, 750004, P.R. China

***** Correspondence: wangzhisheng8239@163.com (Z.W.); changcaibai@163.com (C.B.)

† These authors contributed equally to this work.

Table S1. Qualitative and quantitative analysis of Eno by UPLC-MS/MS.

Compound	Sample content (μg/g)
cyanidin-3-O-glucoside	9026.22
peonidin-3-O-glucoside	2032.35
delphinidin-3,5-O-diglucoside	85.46
delphinidin-3-O-rutinoside	6.91
cyanidin-3,5-O-diglucoside	19.07
pelargonidin-3-O-glucoside	11.21

* Given that Eno is a mixture rich in anthocyanins, to deeply explore the anthocyanin components of Eno, this study employed ultra-performance liquid chromatography-tandem mass spectrometry (UPLC-MS/MS) to detect its main components and their contents. The detection results showed that Eno has six components, among which anthocyanin-3-O-glucoside has the highest content, reaching 9026.22 micrograms per gram.
